# Wege für bessere Impfraten: Indikationsimpfungen bei Erwachsenen

**DOI:** 10.1007/s00108-025-01857-w

**Published:** 2025-02-11

**Authors:** Jürgen Floege, Toralf Schwarz, Christoph Wanner, Uwe Heemann, Baptist Gallwitz, Oliver Witzke

**Affiliations:** 1 Medizinische Klinik II, Uniklinik RWTH Aachen, Pauwelsstraße 30, 52074 Aachen, Deutschland; 2 Praxis für Innere Medizin – Diabetologische Schwerpunktpraxis Zwenkau, Zwenkau, Deutschland; 3 Deutsches Zentrum für Herzinsuffizienz, Julius-Maximilians-Universität Würzburg, Würzburg, Deutschland; 4 Abteilung für Nephrologie, TUM Universitätsklinikum Klinikum rechts der Isar in München, München, Deutschland; 5 Medizinische Klinik IV, Universitätsklinikum Tübingen, Tübingen, Deutschland; 6 Klinik für Innere Medizin, St. Josef Krankenhaus Werden, Universitätsmedizin Essen, Essen, Deutschland

**Keywords:** COVID-19, Hepatitis B, Herpes Zoster, Impfmanagementsystem, STIKO-Empfehlung, COVID-19, Hepatitis B, Herpes Zoster, Vaccination management system, STIKO recommendation

## Abstract

Impfungen stellen eine wichtige Präventionsmaßnahme gegen Viruserkrankungen dar und haben seit ihrer Einführung schon viele Menschenleben gerettet. Heutzutage kann jeder Arzt bzw. jede Ärztin eine Impfung verabreichen. Bei Patienten mit chronischen Erkrankungen sollte eine Indikationsimpfung erfolgen. Generell bietet es sich an, wenn möglich, eine Koadministration vorzunehmen, die in der Regel bei vielen Impfstoffen, insbesondere Totimpfstoffen, problemlos möglich ist. Ein entsprechendes Qualitätsmanagement vermag Komplikationen zu vermeiden. Wichtig ist es auch, die Patienten über Impfungen zu informieren und sie ggf. nach der ersten Impfung an ihren Zweittermin zu erinnern, um die maximale Wirksamkeit zu gewährleisten.

## Abstract (en)

Vaccinations are an important preventive measure against viral diseases and have saved many lives since their introduction. Nowadays, any doctor can administer a vaccination. Patients with chronic diseases should be vaccinated according to indications. In general, it is advisable to carry out co-administration, which is usually possible without any problems with many vaccines, especially inactivated vaccines. Appropriate quality management can avoid complications. It is also important to inform patients about vaccinations and, if necessary, to remind them of their second appointment after the first vaccination in order to ensure maximum effectiveness.

Impfungen stellen eine der wichtigsten Präventionsmaßnahmen in der Medizin dar. Seit 2020 dürfen grundsätzlich alle Ärztinnen und Ärzte Impfungen durchführen, wie durch das Masernschutzgesetz festgelegt (§ 20 Abs. 4 IfSG). Jeder Arzt sollte daher den Patientenbesuch zur Überprüfung und Vervollständigung des Impfschutzes nutzen.

Schätzungen zufolge haben COVID-19-Impfungen die Zahl der pandemiebedingten Todesfälle um mindestens 57 % reduziert und damit in der WHO-Region Europa über 1,4 Mio. Menschenleben gerettet [19]. Es ist eine wichtige ärztliche Aufgabe, in der individuellen Patientenversorgung für einen ausreichenden und umfassenden Impfschutz zu sorgen. Ziel sollte es dabei immer sein, das Impfen einfacher zu machen und praktische Barrieren abzubauen [1].

## Impfbereitschaft nimmt zu

Eine mangelnde Impfbereitschaft zählt zu den wichtigsten Barrieren. Hieß es 2012 noch „Deutschland sucht den Impfpass“, so hat sich jedoch die Einstellung zehn Jahre später möglicherweise pandemiebedingt geändert. Die Bundeszentrale für gesundheitliche Aufklärung konnte in der zweijährlichen Forsa-Befragung mit 5000 Personen im Jahr 2022 nachweisen, dass mehr als vier Fünftel der 16- bis 85-Jährigen (83 %) Impfungen inzwischen befürwortend (56 %) oder eher befürwortend (27 %) gegenüberstehen [6]. 2012 lag dieser Wert mit insgesamt 61 % noch deutlich niedriger.

Dabei zeigten sich signifikante Unterschiede in der Impfbereitschaft zwischen verschiedenen Bevölkerungsgruppen, einschließlich solcher mit Migrationshintergrund oder unterschiedlichen Bildungsabschlüssen. Die größte Akzeptanz bei den Befragten besitzen die Standardimpfungen gegen Tetanus (97 %), Kinderlähmung (92 %) und Masern (87 %) sowie die seit Ende 2020 verfügbare Corona-Schutzimpfung (83 %). Dagegen gab nur die Hälfte der chronisch Kranken (50 %) und der Senioren ab 60 Jahren (58 %) an, sich regelmäßig jedes Jahr gegen die Grippe impfen zu lassen [6].

## Indikationsimpfungen bei Erwachsenen noch verbesserungsfähig

Die Rationale der Indikationsimpfungen bei Erwachsenen zielt in erster Linie auf den Schutz des Einzelnen und erst in zweiter Linie auf die Herdenimmunität. Insbesondere Menschen mit chronischen Erkrankungen (wie Diabetes, chronische Niereninsuffizienz [CKD], Asthma, chronisch-obstruktive Lungenerkrankung [COPD], Herzinsuffizienz, maligne Erkrankungen) sollten vor Erregern geschützt werden, da sie das Immunsystem schwächen und das Risiko für schwere Verläufe erhöhen. Hinzu kommt die fortschreitende und degenerative Veränderung des Immunsystems beim Altern (Immunseneszenz) und gegebenenfalls eine immunsupprimierende Therapie. Zu den wichtigsten Indikationsimpfungen bzw. Grundimmunisierungen zählen in diesem Zusammenhang die Impfungen gegen:

- Influenza
- Pneumokokken
- Hepatitis B
- COVID-19
- Herpes Zoster
- Respiratorisches Synzytialvirus (RSV)

Die vom Robert-Koch-Institut 2022 ermittelten Impfquoten bei der Indikationsimpfung gegen Influenza liegen bei 35,4 % bei Erwachsenen mit impfrelevanten Grunderkrankungen [12] unterhalb des von der Europäischen Kommission festgelegten Impfratenziels von 75 %. Andere Indikationsimpfquoten schnitten in der RKI-Analyse schlechter ab: 25,6 % erhielten innerhalb der letzten sechs Jahre bis 2022 eine Impfung gegen Pneumokokken. Bei 11,5 % der Deutschen über 60 Jahren wurde innerhalb von drei Jahren eine erste Impfung gegen Herpes Zoster durchgeführt und nur bei 7,7 % dann auch die Impfserie mit der zweiten Impfung abgeschlossen.

Seit August 2024 empfiehlt die STIKO die einmalige RSV-Impfung als Indikationsimpfung für Menschen mit schweren chronischen Grunderkrankungen zwischen 60 und 74 Jahren sowie für Bewohner von Pflegeeinrichtungen [10].

### Merke.

RSV-Indikationsimpfung wird bei Menschen mit schweren chronischen Erkrankungen empfohlen.

## Identifizierung geeigneter Patienten

Es sollte im Interesse der impfenden Praxis sein, alle Patienten vor impfpräventablen Infektionen zu schützen. Zunächst gilt es dabei eine Auswahl der Patienten nach chronischen Grunderkrankungen und Risiko zusammenzustellen, indem man im Praxisverwaltungssystem (PVS) nach diesen filtert. Hierzu gibt es in der Literatur konkrete Prozessbeschreibungen, die auch eine Priorisierung zur Influenzaimpfung vorstellen [16].

Eine zweite Möglichkeit stellen kostenpflichtige CE-konforme und EDV-gestützte Impfmanagementsysteme dar, die über eine Schnittstelle an das bestehende PVS angebunden sind [15]. Die Software erhebt bereits beim Öffnen der elektronischen Patientenkartei den Impfstatus und kann den Patienten anhand der Vorerkrankungen stratifizieren. Die Impflücken werden durch ein Ampelsystem angezeigt, und eine Impfplanung wird vorgeschlagen. In der Literatur werden weitere Onlinehilfen genannt, wie z. B. https://www.impfterminmanagement.de/.

## Digitale Impfmanagementsysteme

Im Jahr 2019 waren bereits 7000 Praxen mit einer Software ausgestattet, die das Impfmanagement der Praxis im gesamten Prozess unterstützt [15]. Es gibt einige Anbieter wie ImpfDocNE (GZIM), Impfmodul (WKB-Systempartner) oder X. Impfen (medatixx) auf dem deutschen Markt. Auch eine internationale Übersichtsarbeit kam zu der Empfehlung, digitale Technologien zur Förderung von Impfungen und zur Steigerung der Impfbereitschaft zu nutzen [4].

Im Impfmanagementsystem werden die aktuellen STIKO-Empfehlungen dargestellt sowie u. a. Beschlüsse des Gemeinsamen Bundesausschusses (G-BA), länderspezifische Empfehlungen, reisemedizinische Angaben sowie Impfvereinbarungen von gesetzlichen Krankenversicherungen und Kassenärztlichen Verbänden. Nach der Impfung werden die entsprechenden Abrechnungsziffern und Diagnosen automatisch an das PVS übertragen. Die Impfmanagementsoftware ermöglicht eine zentrale Speicherung von Impfstatus, Terminvereinbarungen und medizinischen Historien. Dies fördert eine effiziente Kommunikation zwischen Fachärzten, da alle relevanten Informationen an einem Ort verfügbar sind. Auch zur Lagerhaltung kann das System beitragen: Warenbestand und ausstehende Lieferungen werden angezeigt, eine Anbindung ans Warenwirtschaftssystem der Lieferapotheke ist möglich. Regeln für ein Minimum an Lagerbestand können vorgeben, ab wann ein Bestellhinweis erfolgt. Allerdings ist die Software kostenpflichtig, mit einmaligen Lizenzgebühren und laufenden monatlichen Kosten.

Die Impfmanagementsoftware wird mittlerweile auch durch einige Krankenkassen gefördert, wie zuletzt in einem Vertrag in der Hausarzt-zentrierten Versorgung vereinbart [5]. Dort wird ein sog. Innovationszuschlag gewährt, wenn bestimmte Anforderungen an das Impfmanagementsystem erfüllt sind (siehe Infobox 1). Auch wenn es sich hier um eine Vereinbarung aus der hausärztlichen Versorgung handelt, stellen die Anforderungen eine gute Richtschnur für andere Praxen dar.

### Infobox 1: Anforderungen an ein modernes Impfmanagementsystem

Der Arzt hält in der Praxis ein digital gestütztes System vor.

- Es erfasst die Impfungen strukturiert und überträgt sie in die jeweilige elektronische Patientenakte, wenn technisch möglich und mit Einverständnis des Patienten.
- Die Software besitzt zumindest die folgenden Funktionen:
  - Überprüfung des Impfstatus nach STIKO-Indikationen
  - Automatische Erstellung von Impfplänen
  - Integriertes Patienteninformationssystem (Merkblätter, Atteste, Aufklärung)
  - Integration aller marktgängigen Impfstoffe
  - Lagerhaltung
  - Rezeptschreibung

Quelle: Aktualisierter Vertrag der Techniker Krankenkasse zur Hausarzt-zentrierten Versorgung [5]. Als zugelassene Software führt der Vertrag die beiden Softwareprodukte ImpfDocNE und X. Impfen auf.

Zum Einfluss der Impfmanagementsoftware auf die Impfrate wird häufig auf eine Studie von 2012 verwiesen [15, 17]. Dabei wurden über 600.000 Impfpasseinträge von 133.559 Impflingen in 110 allgemeinmedizinischen Praxen vor und nach der Implementierung der Impfsoftware verglichen. Zur Auswertung kamen nur die als vollständig dokumentiert gekennzeichneten elektronischen Impfpässe von Personen mit bestimmten chronischen Erkrankungen (Diabetes mellitus, COPD, Asthma bronchiale und koronare Herzkrankheit [KHK]).

Zu Beginn der Softwarenutzung wurde eine Influenzaimpfquote in der Altersgruppe von 18–60 Jahren von durchschnittlich 12–16 % ermittelt, je nach ausgewerteter chronischer Erkrankung. Diese Quote stieg im ersten Jahr auf 32–52 % und im weiteren Verlauf der Jahre sogar über 70 % an. In der Altersgruppe über 60 Jahren nahm die Impfrate zu den genannten Zeitpunkten von 19–23 % über 65–67 % im ersten Jahr sowie im weiteren Studienverlauf auf 85 % und mehr zu. Auch wenn keine Kontrolldaten zu Impfraten in Praxen ohne Impfmanagementsoftware für diesen Zeitraum vorliegen, kann angenommen werden, dass die Steigerung dieser Impfquoten durch das regelmäßige Erinnern des Praxispersonals und der Patientinnen und Patienten sowie durch ein allgemein erhöhtes Präventionsbewusstsein erreicht werden konnte. Denn die eingangs beschriebenen allgemeinen Influenzaimpfquoten liegen durchweg niedriger.

## Motivierung der Patienten

Impfmanagementsysteme können automatisiert alle fälligen Impfungen erfassen und auch Einladungen auf unterschiedlichen Wegen generieren (Brief, E‑Mail, SMS, Telefonliste; [15]). Wie eine qualitative Studie zur Relevanz von digitalen Recall-Systemen mit Kinder- und Jugendmedizinern in und um Bremen ermittelte, liegt der größte Nutzen dieser Systeme bei den Patienten selbst und rechtfertigt den erhöhten Aufwand etwa durch den Datenschutz [14]. Weitere Anreize seitens der Krankenkassen könnten die Verwendung und nachfolgend Akzeptanz der Systeme erhöhen.

Grundsätzlich sollte nicht nur vor Beginn der Wintersaison jeder geeignete Patientenkontakt genutzt werden, um das Thema Impfen anzusprechen. Optionen beinhalten den Erstkontakt, Routinetermine bzw. Termine im Rahmen der Disease Management Programme (DMP), anstehende Reisen oder Auslandsaufenthalte, Lebensveränderungen oder auch einfach im Rahmen von Routineuntersuchungen wie CheckUp35. Denn ein adäquater Impfschutz für Menschen mit geschwächtem Immunsystem ist eine Ganzjahresaufgabe.

Ein kritischer Punkt ist die Erinnerung des Patienten an den Impftermin. Gerade im Falle von Impfserien besteht das Risiko, dass diese nicht beendet werden. Zwar kann der Schutzeffekt meist problemlos durch Nachimpfen verbessert werden, ohne dass serologische Kontrollen erforderlich sind (Hepatitis B sei hier ausgenommen), aber die Zusatztermine sind für beide Seiten ungewünscht. Grundsätzlich scheint eine telefonische Erinnerung durch die Praxis den besten Effekt auf die Einhaltung der Impftermine zu besitzen, wie ein Cochrane Review 2018 beschrieb: Erinnerungs- und Weidereinbestellungs-Interventionen konnten dabei die Immunisierungsrate signifikant und im Mittel um 11 % steigern [7].

## Impfdokumentation wird digitalisiert

Zwei wichtige Elemente für eine gelungene Einbindung der Patienten sind das Pflegen der Impfnachweise und die Erinnerung an den nächsten Impftermin. Noch 2019 besaß jeder Achte in Deutschland keinen Impfpass, unter den Alleinlebenden sogar jeder Fünfte [3]. Grundsätzlich ist eine fehlende Impfdokumentation kein Grund, auf empfohlene oder notwendige Impfungen zu verzichten, denn Impfungen bei eventuell bereits bestehendem Impfschutz haben in der Regel kein erhöhtes Risiko.

Es ist anzunehmen, dass das gelbe Impfdokument sukzessive durch digitale Versionen verdrängt wird, insbesondere nach Einführung der elektronischen Patientenakte (ePA) Anfang 2025. Sie soll die Digitalisierung im deutschen Gesundheitssystem weiter verbessern und auch einen eImpfpass umfassen, dessen Integration jedoch noch einige Zeit in Anspruch nehmen wird. Die zügige Integration des eImpfpasses in die ePA ist jedoch von großer Bedeutung für ein erfolgreiches Impfmanagement.

### Merke.

Die zügige Integration des eImpfpasses in die ePA ist für ein erfolgreiches Impfmanagement von großer Bedeutung.

Laut KBV haben Versicherte dann bei jeder Impfung die Wahl zwischen einem Eintrag im Papier-Impfpass und der digitalen Variante – beides gleichzeitig ist momentan nicht vorgesehen [8]. Für den Zeitraum bis zur Einführung des eImpfpasses sollten/müssten weitere Möglichkeiten zur korrekten Impfdokumentation vorliegen, um einen Informationsverlust zu vermeiden. Neben dem PVS stellt Impfmanagementsoftware eine gute Alternative dar und wird den Datenaustausch mit der ePA über entsprechende Schnittstellen anbieten. Zusätzlich erleichtert sie den Austausch zwischen verschiedenen Arztpraxen. Und auch der entsprechende Datentransfer mit Patienten kann über die Praxissoftware erfolgen – wie im Falle der kostenfreien App ImpfpassDE, die einen datensicheren Transfer aus der ImpfdocNE-Software der Arztpraxis zum Rechner oder Smartphone der Patienten ermöglicht.

## Einbindung des Praxisteams

Impfen ist eine Teamaufgabe. Der/die medizinische Fachangestellte (MFA) nimmt im Impfmanagement der Praxis eine zentrale Rolle ein. Bis auf Indikationsstellung, körperliche Untersuchung und die finale Unterschrift können alle impfrelevanten Tätigkeiten prinzipiell von dieser Person durchgeführt werden. Hierzu zählen etwa die Bestandskontrolle und Bestellung der Impfstoffe, das praktische Impfmanagement, gegebenenfalls die Injektion, sowie im Nachgang die Aktualisierung des Impfpasses. Entsprechend trainierte Mitarbeiter können bei der Terminvergabe oder bei der Rezeptübergabe eine Impfpassprüfung anbieten und evtl. Impferinnerungen mitgeben. Auch im Gespräch oder während einer technischen Untersuchung (z. B. EKG, Spirometrie) kann die Impfprävention thematisiert werden. Sind Impfungen im Rahmen des Praxisbesuches geplant, können MFA alle Vorbereitungen treffen. Dabei bietet die Einrichtung von Impftagen der Praxis die Möglichkeit, Impfung und Dokumentation gebündelt durchzuführen.

Diese Tätigkeiten werden im Rahmen des von Ärzteverbänden anerkannten Fortbildungsgangs zur Impfassistent*in geschult. Im Netz finden sich darüber zahlreiche zertifizierte Schulungen kommerzieller Anbieter und Industriepartner.

**Abb. 1:**
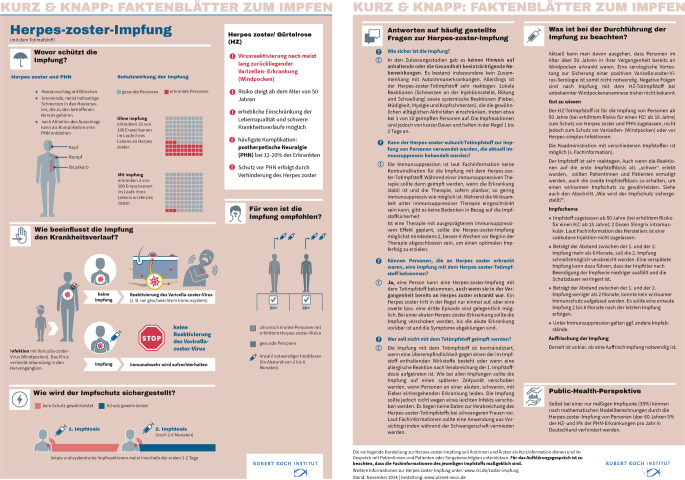
RKI-Faktenblatt zur Herpes-Zoster-Impfung, Stand Februar 2024 [13]. Faktenblätter sind u. a. zu den Infektionserregern Influenza, Pneumokokken, Herpes Zoster, Frühsommer-Meningoenzephalitis (FSME) verfügbar sowie zu vielen anderen Impfthemen

Im Rahmen der Schulung erworbenes Informationsmaterial kann praxisintern zur Fortbildung zuarbeitender Personen genutzt werden. Auch das Robert Koch Institut (RKI) besitzt eine ausführliche Webseite, die sich primär an ein Fachpublikum richtet, aber für interessierte Laien ab mittlerem Bildungsniveau recht verständlich ist. Neben den regelmäßig aktualisierten Ratgeberseiten, die die Informationen nach Infektionserreger bündeln, hat das Institut mit den sog. RKI-Faktenblättern auch druckfähige Abgabematerialien entwickelt (Abb. 1; [13]). Diese zweiseitigen Dokumente könnten in der Praxis ausgedruckt vorliegen und dem geeigneten Patienten vom qualifizierten medizinischen Fachpersonal erklärt und ausgehändigt werden. Informationsmaterialien für Laien mit niedrigem Bildungs- und Sprachniveau können stellenweise über die Impfstoffhersteller erhalten werden – diese bieten gute Flyer oder Online-Materialien an, die den Anforderungen des Heilmittelwerbegesetzes genügen. Zuletzt hat das RKI auch eine eigene App herausgebracht, die kostenfrei von den gängigen App Stores heruntergeladen wird.

## Mehr Effizienz beim Impftermin

Geschulte MFA können die Planung und den eigentlichen Impftermin unterstützen. Impfen ist vor allem eine Zusatzleistung, getreu dem alten Motto: „Niemand kommt nur wegen einer Impfung zum Arzt.“ Daher gilt es vor allem die Zahl der notwendigen Praxisbesuche auf ein Minimum zu reduzieren und niederschwellige Angebote im Rahmen anderer Untersuchungen zu machen. Chronisch Kranke, die zu geplanten Kontroll- oder Behandlungsterminen erscheinen, sind oft auch offen für vorsorgliche Impfungen. Ein probates Mittel zur Effizienzsteigerung ist die Kombination und Koadministration von Impfstoffen. Für Standardimpfungen und ihre Auffrischungen stehen mittlerweile Zwei- bis Sechsfachkombinationen zur Verfügung. Im Falle der Indikationsimpfungen ist dagegen die Koadministration Mittel der Wahl.

### Merke.

Niemand kommt nur wegen einer Impfung zum Arzt.

Für viele Praxen und Patienten bildet die Influenza-Vakzinierung im Herbst den *Taktgeber* der Impfaktivitäten. Die Impfstoffanbieter sind daher bestrebt, die Kombination bzw. Koadministration ihrer Impfstoffe mit einem Influenzavakzin zu validieren, gerade wenn dies aus saisonalen Gründen sinnvoll ist (Infobox 2). Andere Impfstoffe, wie etwa der adjuvantierte Totimpfstoff gegen Herpes Zoster, können zusammen mit Auffrischimpfungen zu anderen Jahreszeiten gegeben werden. Auch die RSV-Impfung wurde bei Erwachsenen im Alter von ≥ 60 Jahren positiv auf die gemeinsame Gabe mit einem standard- oder hoch dosierten saisonalen Grippeimpfstoff überprüft [2] und kann zur Wintersaison mit der Influenzaimpfung gleichzeitig verabreicht werden, vorzugsweise in andere Gliedmaßen.

### Infobox 2: STIKO-Empfehlungen zur Koadministration/Kombination von Indikationsimpfungen

Pneumokokken: Zwischen der Pneumokokken-Impfung und der Verabreichung anderer sog. Totimpfstoffe (z. B. gegen COVID-19 und/oder Influenza) muss kein Impfabstand eingehalten werden. Die Impfungen können beim selben Termin an verschiedenen Gliedmaßen verabreicht werden

- COVID-19: Sofern eine Indikation vorliegt, kann am selben Termin auch gegen saisonale Influenza und Pneumokokken geimpft werden.
  - Herpes Zoster: Die Vakzinierung kann mit anderen Impfstoffen koadministriert werden, wie Tdap (Tetanus, Diphtherie und Keuchhusten), PCV oder dem Influenza-Vakzin, vorzugsweise in verschiedene Gliedmaßen. (Quelle: [11])

Werden mehrere Impfungen in einer Sitzung gleichzeitig verabreicht, wird jede Impfung einzeln abgerechnet. Generell gilt für die Abrechnung von Impfleistungen, dass sowohl die Kodierung zwischen den kassenärztlichen Vereinigungen variieren kann, und ebenso die Vergütung. Auch die Einzelkodierung für eine allgemeine Impfberatung gibt es nicht in allen Regionen. Per Freitextsuche in der entsprechenden Software kann die Kodierung der Abrechnungsziffer ermittelt werden.

### Qualitätsmanagement in der Praxis

Beim Impfmanagement in der Praxis greifen verschiedene Räder ineinander. In der Fachliteratur gibt es Belege für eine Verringerung der Morbidität nach Verbesserung der Qualität. Eine Arbeit zum Qualitätsmanagement aus dem Jahr 2014 identifizierte verschiedene Maßnahmen zur Verbesserung der Qualität ([18]; Tab. 1).

**Tab. 1:** Qualitätskriterien beim Impfmanagement. (Mod. nach [18])

Patientenbezogen	Personalbezogen
Ausreichende Informationen für die Patienten	Impfindikationen und Auswahl der relevanten Impfstoffe gemäß den Leitlinien
Einholung der Patienteneinwilligung	Impfstoffanwendung durch spezialisiertes Personal
Erhöhung der Impfraten durch häufige Kontrolle des Impfstatus und ggf. Angebot von Folgetermin für die nächste Impfung	Qualifikation des Personals (Arzt/Ärztin und/oder die medizinische Assistenzkraft hat innerhalb der letzten zwei Jahre an einer Fortbildung zum Thema Impfen teilgenommen)
*Impfstoffbezogen*	*Lagerungsbezogen*
Standardimpfungen anbieten: Tetanus, Diphtherie, Poliomyelitis, Pneumokokken, Influenza (Herpes Zoster, RSV, SARS-CoV‑2, Pertussis^a^)	Lagergerät: Es wird ein separater Kühlschrank verwendet
Impfstoff-Vorauswahl durch eine Person	Temperatur: Ein Lagerungstemperaturprotokoll wird geführt
Dokumentation der Impfungen in der Patientenakte: Chargennummer, Dosis, Handelsname	Regelmäßige Kontrolle der Impfstofflagerung bezüglich der Kriterien Verpackung, Verfallsdatum und Temperatur

^a^Diese Impfungen waren zum Zeitpunkt der Veröffentlichung noch nicht empfohlen/verfügbar und wurden ergänzt

Mehr Informationen zum Qualitätsmanagement bei Vakzin und Lagerung können in der angesprochenen Publikation oder bei Schelling et al. gefunden werden [15, 18].

Auf dem Fortbildungsportal „Mein Praxischeck“ der Kassenärztlichen Bundesvereinigung wird darüber hinaus ein Online-Kurs „Selbstbewertung Impfen“ angeboten, der von Behandler und Impfassistenten durchgeführt werden kann [9]. Dort lässt sich der Stand des eigenen Qualitätsmanagements anhand von 11 Fragen überprüfen, wie Patientenansprache, -erinnerung, -aufklärung und -information, Impfdokumentation, Verantwortlichkeit, Impfstoffbeschaffung und -lagerung, Abrechnung von Impfleistungen, Erfassen und Melden von Impfkomplikationen und Verdachtsfällen zu Impfschäden, Schutzimpfungen des Praxisteams und Fehlermanagement.

## Sollten Spezialisten impfen?

Um das Impfen einfacher zu machen und Barrieren abzubauen, sollten sich auch Spezialisten überlegen, Indikationsimpfungen in ihrer Praxis durchzuführen. Patienten sind eher bereit, Impfungen zu akzeptieren, wenn sie von einem Arzt kommen, den sie kennen und dem sie vertrauen [1]. Und sie sind eher bereit, sich impfen zu lassen, wenn dies während ihrer regulären Besuche angeboten wird. Daher kann es durchaus vorteilhaft sein, wenn die geübte Schwerpunktpraxis die Vakzinierung durchführt.

Indikationsimpfungen sind heutzutage sicher, effektiv und leicht anwendbar

Benötigt wird dafür neben den angesprochenen Maßnahmen für ein gutes Impfmanagement in der Praxis eine sektorenübergreifende Kommunikation auf Augenhöhe sowie Verantwortungsbewusstsein auf beiden Seiten. Gerade die angesprochenen digitalen Möglichkeiten wie Impfmanagementsoftware und eImpfpass könnten zukünftig dazu beitragen, diesen Informationsaustausch zwischen den Ärzten zu fördern.

Die Zeiten dafür sind günstig. Indikationsimpfungen sind heutzutage sicher, effektiv und leicht anwendbar. Die Digitalisierung im Gesundheitswesen wird das Impfmanagement und die Kooperation unter den Fachärzten weiter verbessern. Wenn sich die Gesellschaft wieder stärker bewusst wird, dass die gemeinsamen Impfanstrengungen eine höhere Mortalität und Morbidität in der Bevölkerung verhindert haben, wird die Relevanz der Impfprävention nicht sinken. Spezialisten sollten daher selbstverantwortlich denken und handeln, sich informieren und dann aktiv werden.

## Fazit für die Praxis

- Jede/r behandelnde Ärztin bzw. Arzt sollte bei seinen Patienten für einen ausreichenden und umfassenden Impfschutz sorgen.
- Menschen mit chronischen Erkrankungen sollten mit Indikationsimpfungen geschützt werden.
- Seit August 2024 wird für Menschen mit schweren chronischen Erkrankungen ab 60 Jahren die Impfung gegen das respiratorische Synzytialvirus (RSV) empfohlen, sowie für Patienten in Pflegeeinrichtungen.
- Zur Effizienzsteigerung sollte die Kombination bzw. Koadministration von Impfstoffen genutzt werden.
- Kostenpflichtige CE-konforme und EDV-gestützte Impfmanagementsysteme können das Impfmanagement einer Praxis unterstützen.
- Bis zum eImpfpass wird noch einige Zeit vergehen.
- Das Robert-Koch-Institut (RKI) bietet auf seinen Webseiten Informationsmaterial an, das auch vom Praxisteam genutzt werden kann.
